# Artificial Intelligence-Aided Diagnosis Solution by Enhancing the Edge Features of Medical Images

**DOI:** 10.3390/diagnostics13061063

**Published:** 2023-03-10

**Authors:** Baolong Lv, Feng Liu, Yulin Li, Jianhua Nie, Fangfang Gou, Jia Wu

**Affiliations:** 1 School of Modern Service Management, Shandong Youth University of Political Science, Jinan 250102, China; lvbaolong2010@sina.com (B.L.); lylfxs@126.com (Y.L.); 2 School of Information Engineering, Shandong Youth University of Political Science, Jinan 250102, China; 3 New Technology Research and Development Center of Intelligent Information Controlling in Universities of Shandong, Jinan 250103, China; 4 Shandong Provincial People’s Government Administration Guarantee Center, Jinan 250011, China; niejianhuasd@126.com; 5 School of Computer Science and Engineering, Central South University, Changsha 410017, China; gff8221@163.com; 6 Research Center for Artificial Intelligence, Monash University, Melbourne, VIC 3800, Australia

**Keywords:** osteosarcoma, artificial intelligence, magnetic resonance imaging (MRI), pre-screening, denoising, edge enhancement, 68T01

## Abstract

Bone malignant tumors are metastatic and aggressive. The manual screening of medical images is time-consuming and laborious, and computer technology is now being introduced to aid in diagnosis. Due to a large amount of noise and blurred lesion edges in osteosarcoma MRI images, high-precision segmentation methods require large computational resources and are difficult to use in developing countries with limited conditions. Therefore, this study proposes an artificial intelligence-aided diagnosis scheme by enhancing image edge features. First, a threshold screening filter (TSF) was used to pre-screen the MRI images to filter redundant data. Then, a fast NLM algorithm was introduced for denoising. Finally, a segmentation method with edge enhancement (TBNet) was designed to segment the pre-processed images by fusing Transformer based on the UNet network. TBNet is based on skip-free connected U-Net and includes a channel-edge cross-fusion transformer and a segmentation method with a combined loss function. This solution optimizes diagnostic efficiency and solves the segmentation problem of blurred edges, providing more help and reference for doctors to diagnose osteosarcoma. The results based on more than 4000 osteosarcoma MRI images show that our proposed method has a good segmentation effect and performance, with Dice Similarity Coefficient (DSC) reaching 0.949, and show that other evaluation indexes such as Intersection of Union (IOU) and recall are better than other methods.

## 1. Introduction

Osteosarcoma is the most common solid tumor of bone origin, accounting for approximately 20% of primary sarcomas of bone [1]. Osteosarcomas frequently occur in adolescents or children under 20 years of age, with more than 75% of patients having an age of onset younger than 25 years [2]. Although some osteosarcomas can be cured by surgical means, some cases remain that are highly fatal even with the most aggressive treatment measures. With the application of comprehensive therapeutic approaches, a cure rate of 65–70% can be achieved in some patients. However, osteosarcoma is prone to lesions with long treatment cycles and poor prognosis, leading to high mortality rates of malignant tumors [3]. Patients with advanced osteosarcoma had a 5-year survival rate of only 20% [4]. The early detection of malignancies can significantly enhance disease cure rates and minimize patient death [5].

In the current application of evaluation of suspected osteosarcoma, radiographs are inaccurate in determining tumor borders, often resulting in results smaller than the actual size of the tumor [6]. Although CT shows the extent of bone destruction well, MRI performs better than CT in showing the extent of focal lesions [7]. MRI has the advantage of providing multidimensional images and allowing for more sensitive quantification of the extent of marrow cavity involvement [8]. Therefore, MRI images are very important for physicians to clinically examine patients with osteosarcoma.

The artificial intelligence-aided detection of medical images is important for predicting patient outcomes and monitoring disease progression with the development of treatment strategies [9,10,11]. Due to the general underdevelopment of health care systems, most countries, especially developing countries, still suffer from a strain and uneven distribution of health care resources [12]. Many hospitals have difficulty in meeting the hardware and staffing requirements for osteosarcoma treatment. Most diagnoses of osteosarcoma rely on the manual recognition of pictures [13]. However, the volume of image data from patients with osteosarcoma is enormous, but few images are of value. Only about 20 of the approximately 700 MRI images generated per patient with osteosarcoma may be useful to the physician for diagnosis [14,15]. The manual screening and processing of validated osteosarcoma images by physicians is a time-consuming and laborious process [16]. Considering the lack of uniformity in the diagnosis of histological features of osteosarcoma, it requires a high level of expertise and knowledge base in pathology on the part of the diagnosing physician. Diagnosis by inexperienced physicians is highly subjective, which can lead to an increased rate of misdiagnosis.

At present, image processing technology is developing rapidly, especially in image segmentation, and new methods are constantly proposed [17]. As medical image processing technology progress, more and more imaging modalities are being employed to diagnose osteosarcoma [18]. Analysis of the extent of tumor infiltration and the boundaries of tumor infiltration by MRI helps clinicians to localize the tumor. MRI images are susceptible to various noise sources (including thermal noise and physiological noise [19] caused by mechanical defects and external signals), which significantly reduces the image quality. The denoising operation is necessary to improve diagnostic efficiency. In addition, osteosarcoma itself has complex local tissue formation and morphological changes, difficult-to-maintain marginal features, and blurred tumor boundaries. Due to this, some medical image processing algorithms are less effective at identifying osteosarcomas, and it is challenging to obtain global multidirectional features and implicit features [20], as well as both accuracy and performance. It is crucial to improve the efficiency of osteosarcoma diagnosis by effectively extracting global features and solving the edge ambiguity segmentation problem without consuming too many computational resources and time costs. Edge feature-based methods are processing models employed in medical images, such as Transformer which has achieved wide application in the field of medical images by taking advantage of global modeling [20,21,22]. Such methods have achieved good results when dealing with simple tasks. When faced with the MRI segmentation task of osteosarcoma where there are a large number of complex boundaries and blurred lesion edges, it is difficult to acquire edge features globally and in multiple directions and to mine the implicit features, making the model accuracy and robustness poor [22]. Therefore, their segmentation results did not meet the expectations.

To improve the recognition accuracy of tumors, this study proposes an artificial intelligence-assisted diagnostic scheme for the MRI images of osteosarcoma with edge-enhanced features. It first processes the raw MRI images using threshold screening filtering (TSF) to filter out the redundant data in lesion-free regions. Then, the fast NLM algorithm and Fourier transform are fused to reduce the noise of the images. Finally, a segmentation network (TBNet) with edge-enhancement features is designed to improve recognition accuracy by enhancing the tumor edge features. It effectively alleviates the problem of the imprecise identification of fuzzy tumor boundaries by existing methods. The network introduces a channeled edge-crossing transformer instead of Unet’s skip connection, and combines loss functions to globally segment tumor regions of different sizes at multiple scales, optimizing the effect of osteosarcoma segmentation.

## 2. Related Work

The segmentation of medical images by computer technology (including CT, MRI, X-ray, etc.), which in turn helps doctors to diagnose diseases, is gradually becoming a popular research topic. Listed and described below are some of the mainstream algorithms in the field.

Recently, visual transformers have been increasingly used for the segmentation of medical pictures. Jienengchen et al. proposed TransUNet [21], the first transformer-based segmentation method in medical imaging. The first transformer-based pure U-shaped design was Swin-Unet [22] proposed by Liu et al. MedT network was proposed by Jeya Maira Jose Valanarasu et al. [23]. To improve medical picture segmentation, Yuhe Gao et al. presented the UTNet [24], a simple and powerful hybrid transformer design that combines self-attention into convolutional neural networks. Yuan Fengji et al. proposed the unified transformer network Multi-Composite Transformer (MCTrans) for cross-scale dependency and semantic consistency learning problems [25]. Olivier Petit et al. combined the complementary capabilities of the U-shaped network and transformer and proposed the UNet-capable of combining the self-attention and cross-attention of both transformers [26]. TransFuse network [27] was suggested by Yundong Zhang et al., which mixes transformers and CNNs in tandem, proposing a fusion of the two techniques, in contrast to most earlier efforts, which replaced the convolutional layers in U-Net networks with the transformer or cascaded the two. HiFormer, a hierarchical multiscale representation transformer was recently proposed by Moein Heidari et al. [28]. Chen Wei et al. presented that HRSTNet [29] replaces the convolutional layer with a transformer module that creates varied resolution feature mapping information. Ruina Sun et al. [30] introduced an effective image classification segmentation technique based on an enhanced Swin transformer, which was designed specifically for lung cancer classification and segmentation. UNeXt model [31] was proposed by Vishal M. Patel et al. as a deep network architecture. CapsNet [32], a capsule network architecture built, was developed by Minh Tran et al. for medical picture segmentation.

In the disease diagnosis of osteosarcoma, image processing by computer technology as an aid to diagnosis has gradually become a research hotspot. The accurate classification of the morphology of different stages of osteosarcoma can enable the timely control of the spread and treatment of osteosarcoma patients in early diagnosis. In the article [33], a CNN has been used to pre-train a publicly available dataset of osteosarcoma tissue images to avoid extensive metastasis. Yu Fu [34] developed a DS-Net network capable of automatically classifying histological images. Hosein Barzekar et al. presented C-Net [35], a new convolutional neural network structure with multiple tandem CNNs. Rahad Arman Nabid [36] proposed a convolutional network to evaluate the grading of patients with osteosarcoma, but the model suffers from the problem of simple overfitting. To identify osteosarcoma cells from osteoblastic cells (MSC), the method proposed by Mario D’Acunto et al. [37] has an accuracy close to one and allows for the study of single cells but requires a huge amount of cellular data. Parlak et al. [38] employed diffusion-weighted imaging to accurately show Ewing’s sarcoma and osteogenic sarcoma by measuring the expression diffusion coefficient (ADC) values for borderline case segmentation of Ewing’s sarcoma and osteogenic sarcoma. 

Many scholars in the literature have proposed the application of many image-processing techniques for the early identification and prediction of treatment options for patients with osteosarcoma. Su Young Jeong et al. [39] proposed the use of machine learning methods incorporating baseline 18-FDG positron emission tomography to predict textural features in scanned images. To this end, Hyung-Jun Im et al. [40] proposed various segmentation methods for pseudo-myelinating lesions, including the relative background threshold method, the gradient-based method (PETedge), and Bluse Otsu (MO-PET). Shuai Limei et al. proposed W-net+ [41], a network structure based on a dense jump connection structure and cascaded dual U-Net. WB Huang et al. [42] proposed a fully automated MRI method for osteosarcoma detection. It is used to identify tumors with irregular structure and shape by using conditional random fields.

The above analysis demonstrates that picture segmentation techniques are becoming increasingly significant in illness diagnosis and prognosis assessment. However, because the pictures are vulnerable to noise, edge features in image segmentation of osteosarcoma are still challenging to retain and segmentation accuracy needs to be improved. In this study, an artificial intelligence-aided diagnosis method for osteosarcoma with edge-enhancement features is proposed, which improves the accuracy of tumor recognition by enhancing edge information.

## 3. Methodology

**Figure 1:**
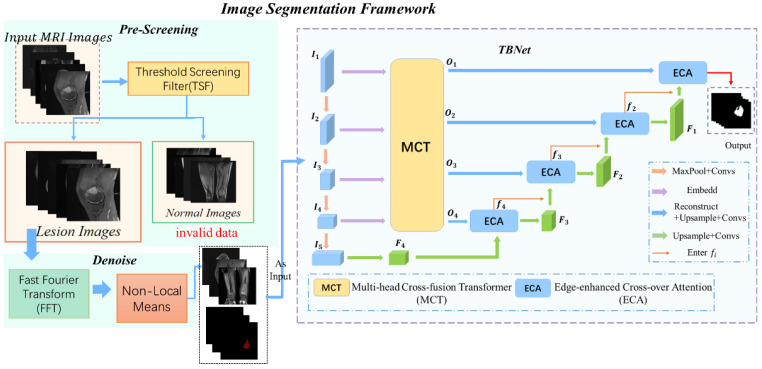
Image Segmentation Framework.

With the development of computer image processing technology, artificial intelligence solutions are widely used in the medical field. Its importance is to solve the problems of high consumption of medical resources and low efficiency of disease diagnosis in developing countries [43,44,45,46]. However, such methods still present major challenges in the recognition of tumors. Taking osteosarcoma MRI images as an example, existing methods have difficulty in handling images with complex lesions and blurred edges [10,47]. This paper proposes an artificial intelligence-aided diagnosis scheme to provide more options for osteosarcoma-aided diagnosis in developing countries. The overall design of the scheme is in Figure 1. First, we perform MRI image pre-screening by Threshold Screening Filter (TSF) to filter redundant data and extract useful images for submission to the next step; then, we introduce a combined fast Fourier transform NLM algorithm for noise reduction in MRI images to improve the diagnostic quality. Finally, to improve the recognition accuracy of the model for tumor edges, a U-shaped network with a fused transformer featuring edge enhancement is used to segment the pre-processed images. The channeled transformer bridges the semantic gap that exists in the UNet network. The method also introduces the edge-enhancement module (BAB) with a combined loss function to optimize the effect of edge segmentation. 

The artificial intelligence-aided diagnosis scheme of the osteosarcoma MRI images we constructed is divided into two main sections: Section 3.1 is data preprocessing; Section 3.2 is the image segmentation model of osteosarcoma MRI. In Section 3.1, we are mainly divided into two parts, which are the pre-screening operation based on Threshold Screening Filter (TSF) and the noise-reduction processing based on a fast NLM. Table 1 shows the main mathematical symbols of this chapter and their annotations.

### 3.1. Data Pre-Processing

#### 3.1.1. MRI Image Pre-Screening Based on Threshold Screening Filter (TSF)

The purpose of pre-screening is to obtain some valuable images from the original MRI images. For MRI image filtering, we used a threshold filter (TSF).

(1) $$T\left(a\right)=k(\left|\frac{\sum_{1\leq i\leq N_{1}}\sum_{1\leq j\leq N_{1}}\left({\bar{B}}_{a}\left(i,j\right)-0.5\lambda_{T}\right)}{n_{1}{}^{2}}\right|-\lambda_{T\;}$$

where $\lambda_{T}$ is a hyperparameter of the TSF controlling the screening threshold of the lesion.

Noise significantly reduces the accuracy and there is no greater need for edge details during pre-screening for the time being. Therefore, we introduced the discrete Fourier transform (DFT) [48] for coarse initial denoising of noise at high frequencies first. ${\bar{B}}_{a}$ is the coarse initial noise reduction method for $B_{a}$.

(2) $${\bar{B}}_{a}=F_{2D}^{-1}(s(F_{2D}(B_{a}),\lambda_{FFT2}\sigma \sqrt{2log(n_{1}^{2})}))$$

where $F_{2D}$ represents the DCT transform operator, $F_{2D}^{-1}$ represents the inverse of $F_{2D}$, and $\lambda_{FFT2}$ is the fixed threshold parameter. Additionally, s is defined as:

(3) $$s\left(a,\lambda \right)=\left\{\begin{array}{c} 0,\;\;\;-\lambda <a<\lambda \\ \lambda ,\;a>\lambda \;,a<-\lambda \end{array}\right.$$

In Equation (1), *k* is:

(4) $$k\left(a\right)=\left\{\begin{array}{c} 0,a\geq 0\\ 1,a<0 \end{array}\right.$$

If the output of *k*(*a*) is 1, the location of the window block $B_{a}$ contains the lesion area, and the image is kept to mark the image as “lesion image”. When it is not 1, the image of window block $B_{a}$ is considered invalid and the sliding traversal of the entire image continues. After the original data set is pre-screened, the redundant data without lesions are eliminated, and the useful and valuable image data are retained for further operations. 

#### 3.1.2. Noise Reduction Based on Fast NLM Algorithm

The degree of tumor invasion and the invasion boundary is different, which affects the recognition effect of the model to a certain extent. The source of noise influence in MRI images is different from the usual images. The usual images are perturbed by Gaussian white noise. MRI images, on the other hand, are influenced by the bias induced by the Rician distribution noise associated with the MR acquisition system signal, as well as Gaussian white noise. Real and imaginary channels are influenced by and collect complex MR data *X*.

(5) $$X=Scos\theta +\eta_{1}+j\left(Ssin\theta +\eta_{2}\right)$$

where *S* is the original MR image and θ is the phase. Changing the noise distribution to Rician can be expressed as:

(6) $$P(\frac{X}{S},\sigma )=\frac{X}{\sigma^{2}}e^{-(\frac{x^{2}+S^{2}}{2\sigma^{2}})}I_{0}(\frac{XS}{\sigma^{2}})$$

By first squaring the magnitude of the data *X* and then considering the expectation of both sides, it is possible to determine the bias caused by the Rician distribution.

The non-local mean (*NLM*) takes advantage of the redundancy of the image, and the pixels in the image filtered by non-local averaging are a weighted average of all other pixels [7]. NLM can successfully remove the aforementioned image noise. This number is determined by examining the picture block and determining the sliding window’s similarity to the selected window. The NLM method’s core equation is Equation (7).

(7) $$NLM\left(I\left(i\right)\right)=\sum_{j\in N_{i}}\omega \left(N_{i},N_{j}\right)I\left(j\right)\left(\omega \left(N_{i},N_{j}\right)=\frac{1}{Z\left(i\right)}e^{\frac{\left(-p\right)}{h^{2}}}\right)$$

where $0\leq \omega \left(N_{i},N_{j}\right)\leq 1,\underset{j\in N_{i}}{\sum }\omega \left(N_{i},N_{j}\right)=1$, $\omega \left(N_{i},N_{j}\right)$ is the weight to calculate the similarity between two patches, h represents the filtering parameter, and p represents the Euclidean distance between two patches.

The original NLM algorithm takes a lot of time to compute the similarity weights and has a high distance computation complexity [49]. To keep the NLM accurate while reducing the consumption of computational resources, we introduce the Fast Fourier Transform (FFT) strategy to implement a fast algorithm for computing pixel-level NLM. *v* is the input noisy image, *V* is the patch, $c_{s}$ is the patch edge half-length, *c* is the patch side length as $c=2\times c_{s}+1$, and $c^{2}$ is the number of pixels in the patch procedure. First, we rearrange the loop to consider all pixels *i* of all translation vectors $t\in {\left[\left[-C_{s},+C_{s}\right]\right]}^{2}$. In the context of patches distance, the Euclidean distance can be calculated as follows:

(8) $$s_{t}\left(i\right)={\left[\left[v\left(i\right)-v\left(i+t\right)\right]\right]}_{2}^{2},i=\left(i_{1},i_{2}\right)\in \Omega$$

Then, in this case, the weighted norm of the patch difference is a discrete convolution:

(9) $${\left|\left|\;V\left(i\right)-V\left(i+t\right)\right|\right|}_{2.K}^{2}=\sum_{\left\{b\in {\mathbb{Z}}^{2}:\left|\left|b\right|\right|\propto \leq d_{s}\right\}}K\left(b\right){\left|\left|v\left(i+b\right)-v\left(i+t+b\right)\right|\right|}_{2}^{2}=\left(\tilde{K}*s_{t}\right)\left(i\right)$$

where  * denotes discrete convolution, $\tilde{K}\left(b\right)=K\left(-b\right)$. It is calculated by F and its inverse $F^{-1}$.

(10) $${\left|\left|V\left(i\right)-V\left(i+t\right)\right|\right|}_{2,K}^{2}=F^{-1}\left(F\left(\tilde{K}\right)F\left(s_{t}\right)\right)\left(i\right)$$

The time complexity of FFT is $O\left(ND^{2}log\left(N\right)\right)$, subject to the computation of any translation vector $t\in {\left[\left[-C_{s},+C_{s}\right]\right]}^{2}$, and the computation of NLM weights is independent of the patch size. Assuming that the total number of image pixels is *N*, the introduction of FFT enables the NLM algorithm to obtain a good speedup. Specifically, the time complexity is reduced from the original $O\left(ND^{2}c^{2}\right)$ to $O(ND^{2}log\left(N\right)$. As shown in Figure 1, after the image data are processed by noise reduction, they are then put into the subsequent segmentation operation.

### 3.2. Tumor Localization

**Figure 2:**
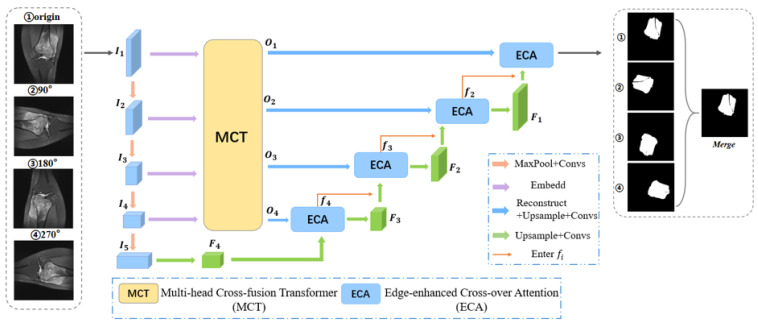
TBNet Segmentation Model.

The original data are input to the segmentation model TBNet for segmentation after a series of data preprocessing, such as image pre-screening and noise reduction. TBNet is a U-Net network with edge-enhanced features of a fusion transformer designed by us, which can effectively solve the edge blur segmentation problem caused by blurred lesion edges. TBNet is based on the UNet network without skip connection, introducing a multi-head cross-fusion transformer (MCT), edge-enhanced cross-attention module (ECA) with combined loss function; this optimizes the segmentation effect and solves the edge blur segmentation problem. The model mainly consists of U-Net without a skip-connection mechanism [50], channel edge cross-fusion transformer (including multi-head cross-fusion transformer module MCT and edge-enhanced cross-attention module ECA), and combined loss function. Figure 2 shows the general design of the model.

The original U-net does not work well in the osteosarcoma diagnosis task. Osteosarcoma itself has complex morphological changes, difficult-to-maintain edge features, and blurred tumor boundaries, coupled with the fact that MRI images, the tools relied on for diagnosis, are vulnerable to noise. This makes the skip connection mechanism have a certain semantic gap in the osteosarcoma segmentation recognition task, makes the stage features incompatible, and makes it difficult to obtain edge features and mine the implicit features globally and in multiple directions, thus making the model accuracy and robustness poor with a certain impact on the segmentation. We introduce a cross-fusion channel transformer with edge-enhancement features to replace the jump connection while taking advantage of the transformer and Unet for the cross-fusion of multi-scale channel information, solving the problem of semantic hierarchical inconsistency, and enhancing edge information to improve the edge segmentation ambiguity problem.

The channelized edge cross-fusion transformer consists of MCT for encoder feature transformation, MCT for encoder feature conversion, and the edge-enhancement cross-attention module (ECA) for decoder feature fusion.

(1) Multi-head cross-fusion transformer (MCT) for encoder feature transformation

**Figure 3:**
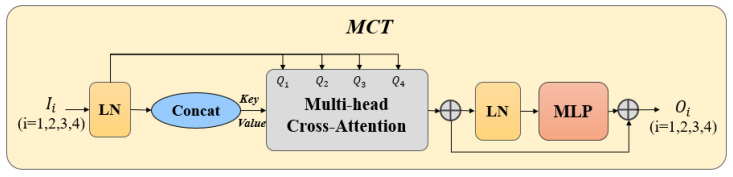
Multi-Head Cross-Fusion Transformer (MCT).

MCT is a multi-scale global feature investigation of osteosarcoma MRI images using transformer long-dependent modeling to fuse multi-scale encoder features. It is divided into three stages: Embedding Multi-scale Features, Multi-Crossing Attention, and Multi-Layer Perceptron. Figure 3 shows the design of the MCT.

**Embedding Multi-Scale Features.** The embedding of multi-scale features starts by labeling the features with the $I_{i}$ of the multi-scale feature of the given osteosarcoma MRI image, and reshaping the 2D patch of the flat feature sequence so that the patch size is P, P/2, P/4, and P/8. In this process, the channel size remains the same. We then connect the four layers as keys and values $L_{\Sigma }=Concat\left(L_{1},L_{2},L_{3},L_{4}\right)$ and feed them into a multi-headed cross-attention module to encode the channels and dependencies to refine the features at the encoder level of each osteosarcoma MRI image using multiscale features.

**Figure 4:**
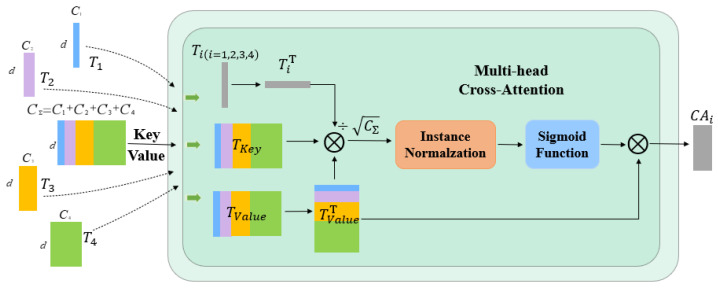
Multi-Head Cross-Attention Module.

**Multi-Crossing Attention.** As shown in Figure 4, there are five inputs required for this module. Let $T_{i}\epsilon R^{C_{i}\times d\;},T_{Key}\epsilon R^{C_{\Sigma }\times d},T_{Value}\epsilon R^{C_{\Sigma }\times d}$, and $W_{i}\epsilon R^{C_{i}\times d\;},\;W_{Key}\epsilon R^{C_{\Sigma }\times d},\;W_{Value}\epsilon R^{C_{\Sigma }\times d}$ be the weights of different inputs; d is the sequence length, $C_{i}\left(i=1,2,3,4\right)$ is the channel size to skip the concatenated layer, and the four sizes we use are $C_{1}=64,C_{2}=128,C_{3}=256,C_{4}=512$. Through the cross-notice mechanism, a similarity matrix $M_{i}$ is generated and $T_{Value}$ is weighted.

(11) $$T_{i}=L_{i}W_{i}\;,T_{Key}=L_{\Sigma }W_{Key},T_{Value}=L_{\Sigma }W_{Value}$$

(12) $$CA_{i}=M_{i}T_{Value}^{Τ}=\sigma \left[\psi \left(\frac{T_{i}^{Τ}T_{Key}}{\sqrt{C_{\Sigma }}}\right)\right]T_{Value}^{Τ}=\sigma \left[\psi \left(\frac{W_{i}^{Τ}L_{i}^{Τ}L_{\Sigma }W_{Key}}{\sqrt{C_{\Sigma }}}\right)\right]W_{Value}^{Τ}L_{\Sigma }^{Τ}$$

where $\psi \left(\cdot \right)$ and $\sigma \left(\cdot \right)$ denote the instance normalization and the softmax function, respectively. We change the original method of the attention operation along the patch-axis in the self-attention mechanism and change the direction to along the channel direction. Instance normalization is also used to normalize each instance’s similarity matrix on the similar mapping, allowing for the gradient to flow smoothly. In the case of N-head focus:

(13) $$MCA_{i}=\frac{\sum_{0<j<N,j\in Z}CA_{i}^{j}}{N}$$

**Multi-Layer Perceptron (MLP).** After the multiple cross-notice mechanisms, the results are fed into the MLP with the residual structure and residual operator to obtain the output. An L-layer transformer is constructed by repeating the operation equation (14) L times. The four outputs $O_{1},O_{2},O_{3},O_{4}$ of the L-th layer are reconstructed by up-sampling the operations after convolutional layers. They are connected to the decoder features $F_{1},F_{2},F_{3},F_{4}$, respectively, and fed to the Edge-Enhanced Cross-Attention (ECA).

(14) $$O_{i}=MCA_{i}+MLP\left(T_{i}+MCA_{i}\right)$$

The segmentation of the focus area in the MRI image is a binary classification task. The introduction of too many layers of transformers will lead to the explosive growth of computational complexity and model over-fitting problems. After balancing resource cost and segmentation accuracy, N and L are set to 4.

(2) Edge-Enhanced Cross Attention (ECA) module for decoder feature fusion

**Figure 5:**
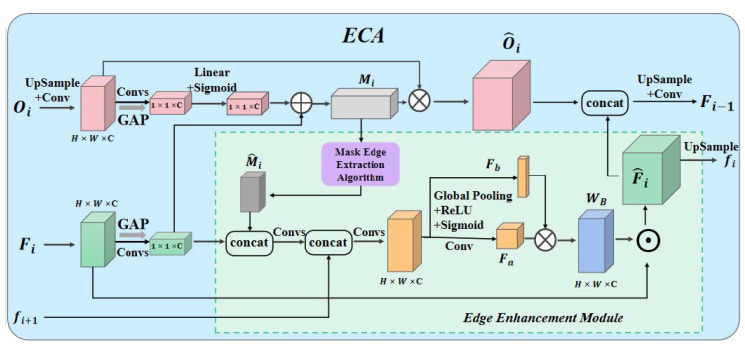
Edge-Enhanced Cross-Attention Module (ECA).

As shown in Figure 5, we propose a channel-based edge-enhancement cross-attention module to better fuse the semantic inconsistency characteristics between the channel transformer and the U-Net decoder, as well as to correct for fuzzy edge segmentation and the lack of some regions in osteosarcoma MRI images. The module is split into two sections: Decoder Channel Crossing Attention Module and Edge-Enhancement Module. It can not only direct the channel and information filtering of interceptor features to minimize ambiguity in decoder features, but it can also increase edge information to replenish missing regions.

**Decoder Channel Crossing Attention Module.** We take level *i* transformer output $O_{i}\in R^{C\times H\times W}\;$and $F_{i}\in R^{C\times H\times W}\;$ as the input for cross-attention. Spatial squeezing is performed through the GAP layer to construct attention masks.

(15) $$M_{i}=X_{1}\cdot \xi \left(O_{i}\right)+X_{2}\cdot \xi \left(O_{i}\right)$$

where the resulting vector $\xi \left(x\right)\in R^{C\times 1\times 1}$, the *k*-th channel $\xi \left(x\right)=\frac{1}{H\times W}\sum_{i=1}^{H}\sum_{j=1}^{W}X^{k}\left(i,j\right)$, $x_{1}\in R^{C\times C}$ and $x_{2}\in R^{C\times C}$. We recalibrate and excite $O_{i}$.

(16) $$\hat{O_{i}}=\sigma \left(M_{i}\right)\cdot O_{i}$$

**Edge-Enhancement Module.** The blurred focus edge in MRI images often leads to inaccurate tumor segmentation. To solve the problem of edge fuzzy segmentation, the edge-enhancement module is introduced into the cross-attention module of the feature fusion part of the encoder.

(17) $$F_{ab}=d_{3}\left[c\left(d_{3}\left[c\left(d_{1}\left[F_{i}\right],\hat{M_{i}}\right),f_{i+1}\right]\right)\right]$$

For the edge attention mechanism, we are inspired by the spatial channel compression and stimulus attention module and designed the attention module that focuses more on the edge feature information with the same simplicity and without increasing the parameters. The specific process can be expressed as follows: first, the input feature map $F_{ab}\in R^{C\times H\times W}$ is compressed in channel and space, and the compressed feature map $F_{a}\in R^{H\times W\times 1}$ obtained after the compression is multiplied with the vector $F_{b}\in R^{1\times 1\times C}$ to obtain a weight $W_{B}\in R^{C\times H\times W}$ of the same size as the input. This not only provides a corresponding weight for each pixel of the input feature map but can also highlight more important location information at the edges and suppress location information of a small value.

(18) $$\hat{F_{i}}=\left(F_{a}\times F_{b}\right)⨀F_{ab}$$

where × represents the expansion to direct multiplication and ⨀ represents the pixel-by-pixel multiplication. Finally, the output $\hat{O_{i}}$ of the decoder channel cross-attention module and the output $\hat{F_{i}}$ of the edge-enhancement module will converge to obtain the final output $F_{i}$. $F_{i}$ will continue to work as an input to the *i* − 1 layer.

In addition, the following loss function is proposed.

(19) $$L=\alpha L_{Dice}+\beta L_{BD}$$

It combines dice loss and boundary loss:

(20) $$L_{Dice}=1-\frac{2PT}{P^{2}+T^{2}}$$

(21) $$L_{BD}=\int_{\Omega }\phi_{G}\left(i\right)g\left(i\right)di$$

where *P* is the predicted value, *T* denotes the true label value, *G* is Ground Truth, and $g\left(\cdot \right)$ is the softmax probability output of the network. If i∈G, then $\phi_{G}\left(i\right)$ is the negative value of the distance between the point and *G*; conversely, it has a positive value. We introduce parameters α and β as balancing coefficients in the defining Equation (19) of the integrated loss function L.

By focusing on both the tumor region and edge information, our method effectively alleviates the edge-blurring problem that has not been addressed in previous research methods.

## 4. Experimental Analysis

### 4.1. Dataset

The data for this article are from the Research Center for Artificial Intelligence of Monash University [51]. The original dataset included more than 4000 MRI image samples from 204 patients with osteosarcoma. All patients are diagnosed by specialists at the hospital based on clinical presentation and pathological images. All images were rotated by 90°, 180°, and 270°, respectively, to improve the generalization ability of the model. Finally, the dataset was partitioned into a training set, a validation set, and a test set in a ratio of 7:1:2. In addition, the ground-truth label of our MRI images was carried out by a collaboration of three doctors at the hospital. As shown in Figure 1, in this mission, only the focal area needs to be segmented. Therefore, the ground-truth label consists of two parts—the tumor area is the foreground and the other part is the background. 

The experiments in this paper were conducted under the Ubuntu 18.04.5 operating system with Python as the main programming language, Python version 3.8, and PyTorch version 1.10.0. The GPU used in the experiments was a GTX 3060, and the CPU used was an Intel(R) Xeon(R) E5-2630L v3 with 30 GB of RAM.

### 4.2. Evaluation Metrics

Based on an earlier study, classification accuracy (*CA*) expresses the ratio between the screened accurate samples and the expected samples [52]. We used CA to assess the validity of the MRI image pre-screening algorithm. Let $C_{R}$ be the correctly classified image, i.e., the lesioned image that is correctly screened out by the normal image, $C_{F}$ be the incorrect classification result, and $C_{R}+C_{F}$ be denoted as all the images of the input. The overall error of this process can be denoted by $C_{F}$.

$$CA=\frac{C_{R}}{C_{R}+C_{F}}$$

The peak signal-to-noise ratio (*PSNR*) can objectively quantify the denoising effect. *NMISE* represents the normalized mean squared error of integration between the denoised and noise-free data [53].

$$PSNR=10{log}_{10}(\frac{max{\left(p_{i}\right)}^{2}}{NMISE})$$

Intersection of Union (*IOU*) is a metric that is commonly used to measure the similarity between the predicted tumor region and the actual tumor region [54]. Specifically, IOU is the ratio of the intersection region between the judged tumor region $I_{1}$ and the real tumor region $I_{2}$. The larger this index is (the closer it is to 1), the greater the impact of the segmentation.

$$IOU=\frac{I_{1}\cap I_{2}}{I_{1}\cup I_{2}}$$

*DSC* is commonly used to indicate segmentation effects on a similarity measure from 0 to 1 [55]. The DSC is calculated as the ratio of twice the area of the junction of $I_{1}$ and $I_{2}$ to the entire area, as expressed in the following equation. The best segmentation effect is obtained when the DSC is 1.

$$DSC=\frac{2*\left|I_{1}\cap \left.I_{2}\right|\right.}{\left|I_{1}\right|+\left|I_{2}\right|}$$

The segmentation network’s performance is explained using a confusion matrix. Among them, TP indicates that the area is predicted and is actually a focus area. TN indicates that both predicted and actual tissues are normal. FP represents the predicted tumor area, which is actually normal tissue. On the contrary, FN indicates that it is predicted to be normal, but it is actually a tumor. In this experiment, accuracy (ACC), precision (Pre), recall (Re), and F1-score (F1) are calculated by using the confusion matrix to measure the performance of each network [56].

### 4.3. Algorithm Comparison

We perform a comparative experimental analysis with our proposed TBNet using the following method. These methods are briefly described below.

(1) A fully convolutional network (FCN) is a pixel-level classification of images, using skip structures to achieve fine segmentation [57]. In this paper, 2 networks with 8 and 16 up-sampling are used FCN-8s and FCN-16s.
(2) The PSPNet focuses on the pyramid pool module as a technique of extracting global contextual information, collecting and fusing contextual information at various scales, thus being particularly effective in acquiring global information [58].
(3) The MSFCN is a fully convolutional network with many supervised lateral output layers for automatic volume segmentation [59]. To enable the effective learning of local and global visual features, a supervised lateral layer is added to the three layers of the convolutional network to provide a system-level structure to guide multidimensional feature learning.
(4) Multi-scale residual networks (MSRN) [60] can adaptively identify image features at different scales and make them interact to produce effective high-resolution picture information. The generation of structural multiscale residual block MSRBs is achieved by combining convolution kernels of different sizes on the basis of the creation of residual blocks.
(5) U-Net is a symmetric U-shaped structure that enables image-semantic-level segmentation [61]. The left side is a convolutional layer (systolic path) responsible for feature extraction and the right side is an up-sampling layer (extended path) responsible for feature reduction. It can use very little data to obtain the best results.
(6) Feature pyramid networks (FPN) [62] use low-level feature semantic information with accurate target locations and information-rich high-level feature semantic information to make predictions independently at different feature layers using multi-scale feature fusion.
(7) The UTNet network [24] hybrid transformer design incorporates self-attention into convolutional neural networks. The self-attention module is used in both the encoder and decoder in UTNet, and it is combined with relative position coding to considerably minimize the complexity of the self-attention process. In addition, a self-attentive decoder is presented to recover fine-grained features from the encoder’s skipped connections, addressing the problem that the transformer requires a considerable quantity of data to acquire the visual sensing bias.
(8) The TransUNet network [21] combines the advantages of a transformer and U-Net in a hybrid CNN transformer design. The global context extraction encodes the tokenized picture blocks in the CNN feature map as input sequences by the transformer. The decoder performs up-sampling to achieve accurate localization. This operation is performed before combining the encoded features with the high-resolution CNN feature map.

### 4.4. Influence of Super Parameters

In the preparation before training, to improve the efficiency of training and model performance, we first performed the pre-screening and noise-reduction operation of the data to avoid meaningless redundant and invalid data, which affect the improvement in segmentation efficiency. For the fixed parameters of the TSF in the pre-screening operation, referring to the general settings of the study, we set $n_{1}$ to 7 and the fixed threshold parameter $\lambda_{FFT2}$ to 0.82.

**Figure 6:**
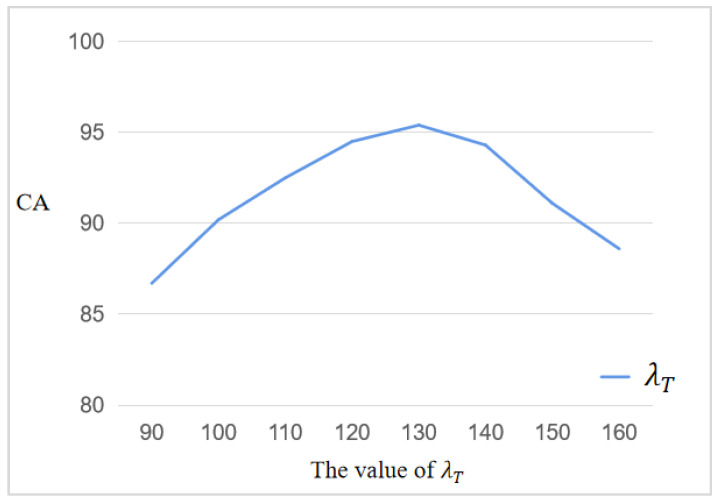
Trend diagram of CA with $\lambda_{T}.$

In addition, for the setting of parameter $\lambda_{T}$ in TSF, we found that the reasonable setting interval of $\lambda_{T}$ should be 90–160 by repeating the experiment and statistically analyzing the lesion areas on the MRI images of different osteosarcomas. If $\lambda_{T}$ is set too small or too large, the accuracy of screening is reduced. The former is because it causes the TSF to incorrectly classify lesioned images as normal, reducing the accuracy of the screening process. The latter is because the TSF will incorrectly label healthy images as lesions. The average classification accuracy is maximum when $120<\lambda_{T}<140$, which may reach 95.82%, and the optimal interval of $\lambda_{T}$ can be defined as 120–140. The trend of classification accuracy (CA) can be shown in Figure 6 for a suitable range of parameters.

### 4.5. Results

From the MRI images screened by the TSF algorithm (the lesion images used for the experiment and the rejected normal images), 100 images were selected separately and the accuracy of the model was judged by the confusion matrix, as shown in Table 2. The predicted values indicate the results judged by the TSF algorithm. The classification results judged by the physician after examining the MRI images are represented by the actual values. Among them, the model correctly classified 93 normal images and lesion images, judged 3 lesion images as normal, and judged 4 normal images as lesion images. According to the analysis in Section 4.4, the best CA value of the TSF algorithm reached above 0.95. The CA value of the randomly selected results in Table 2 reached 0.93. It proves that the TSF algorithm is as expected and can effectively eliminate redundant data from lesion-free regions and filter out valuable training images.

**Figure 7:**
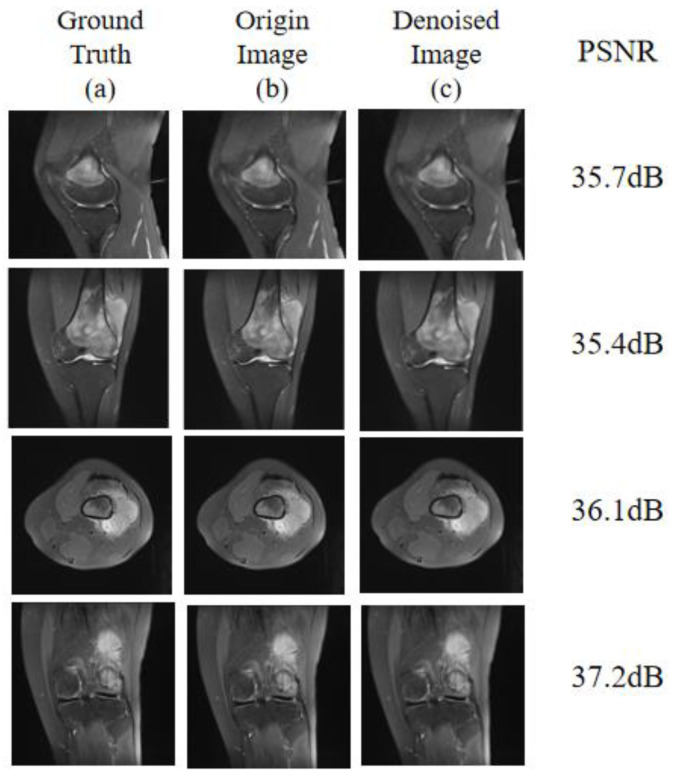
(**a**) Noise-free image (ground truth), (**b**) origin image before noise reduction, (**c**) reconstructed image after noise reduction.

To verify the effectiveness of the denoising process, we used the evaluation metric PSNR to quantify the validity of this operation. Figure 7 shows the reconstruction results of the images before and after noise reduction with the corresponding PSNR values. By comparison, it can be observed that the post-noise-reduction image (Figure 7b) is clearer than the image before the noise-reduction treatment (Figure 7a) and is very close to the noise-free image (Figure 7c). Meanwhile, we can know that the PNSR value will be higher after the noise-reduction processing.

**Figure 8:**
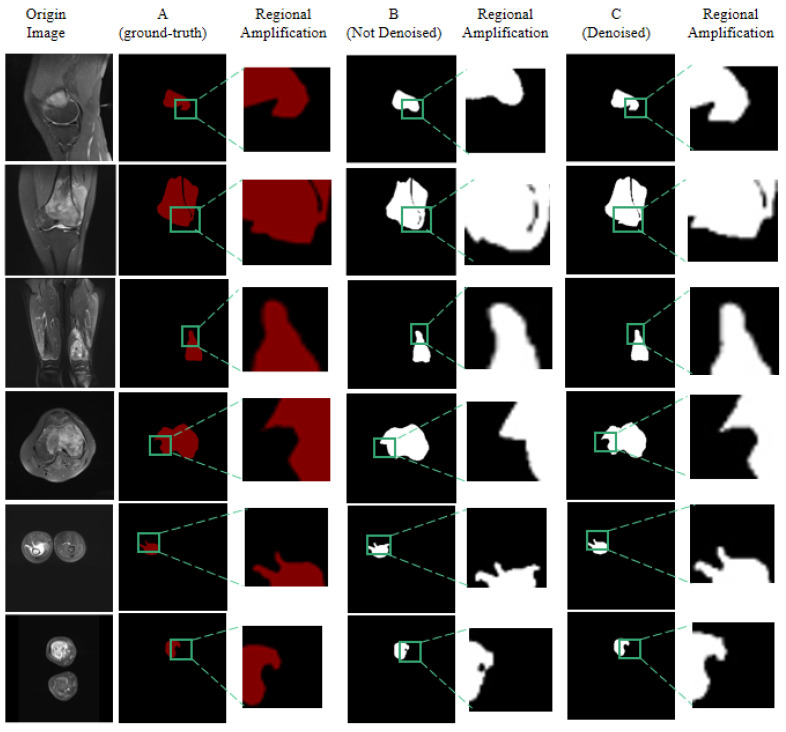
The segmentation effect before and after noise reduction. The sub-figure (**A**) represents the ground truth of the image, the sub-figure (**B**) represents the result of image segmentation without Denoised, and the sub-figure (**C**) represents the result of image segmentation with Denoised.

Specifically, Figure 8 shows the effect comparison of some randomly selected images before and after the noise-reduction process to prove that noise reduction has a great impact on the recognition accuracy of tumors. Comparing the segmentation effect before noise reduction (Figure 8B) and after noise reduction (Figure 8C), the segmentation effect is similar in the global view. However, we can visually see from the details of the local area magnification that the segmentation effect after noise reduction (Figure 8C) compensates for the detailed contour of the edge area of the lesion compared with the segmentation effect without noise reduction (Figure 8B), which is closer to the real image. The results demonstrate that noise-reduction processing increases accuracy.

In Table 3, we further visualize the effect of the TSF filtering algorithm $\left(\lambda_{T}=133\right)$ and the NLM noise-reduction algorithm on the performance of the model. When the original MRI images were processed directly by the TBNet network, all the other indexes performed poorly, although its Re value reached 0.974. When images without tumor regions (or tumor regions barely visible) were removed using the TSF method, the performance of our model improved more significantly. Precision increased by around 0.009 after image pre-screening, F1 increased by about 0.003, recall increased by about 0.001, and DSC increased by about 0.006. The most important DSC index improved by roughly 0.012 after the noise-reduction treatment, whereas precision, F1, and IOU improved by 0.005, 0.002, and 0.011, respectively.

**Figure 9:**
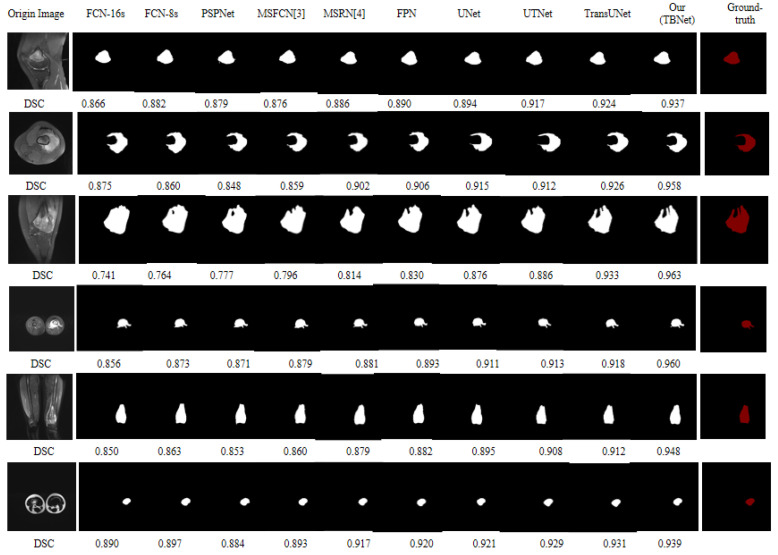
Comparison of different models for the segmentation of MRI images of osteosarcoma.

After the screening of the dataset by Threshold Screening Filter (TSF), more than 3000 MRI images remained. All comparison methods, including UNet, were experimented with using the pre-processed images to ensure fairness. The results of each model for the segmentation of osteosarcoma MRI images are shown in Figure 9 below. We can visually examine the model’s segmentation performance using ground-truth photographs. Meanwhile, we have selected the DSC metrics, and based on the following six segmentation examples of osteosarcoma, we can find that the TBNet network can obtain better segmentation results. When dealing with simple segmentation jobs, our technique can obtain the same segmentation outcomes as previous models. It performs better when dealing with tumor segmentation jobs with complex boundaries (cases 3 and 4). Our method, particularly in the specifics of the border contour of the osteosarcoma lesion, can obtain a more precise and comprehensive segmentation of the tumor borders with higher accuracy when compared to other methods that can handle it appropriately.

**Figure 10:**
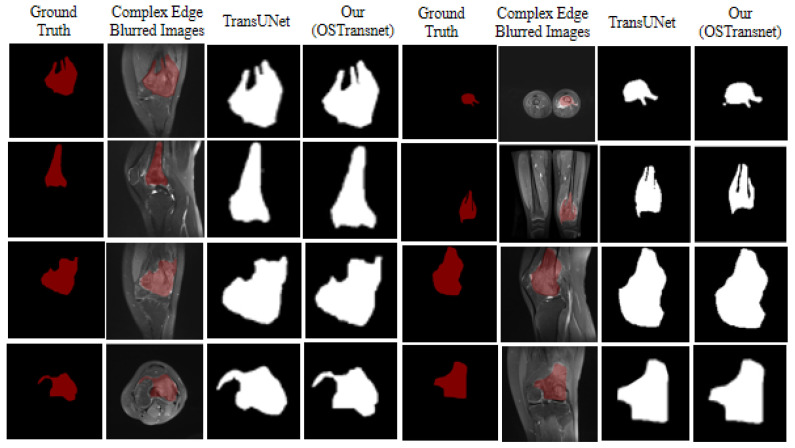
Segmentation effect with blurred edges.

Similarly, for the same osteosarcoma images with complex edge blurring characteristics as in cases 3 and 4 above, we selected the following six examples of osteosarcoma images with blurred edges and used TransUNet, and our proposed TBNet for image segmentation, respectively. As shown in Figure 10, our method can segment the boundary of the lesion region more effectively and precisely.

We measured segmented data to quantitatively compare the effectiveness of the various methods by comparing several classical comparative metrics. The osteosarcoma task is shown in Table 4. Our proposed model TBNet performs well overall in the task. Several metrics of our scheme are the highest. For example, ACC, IOU, DSC, and Re reach 0.997, 0.915, 0.949, and 0.969, respectively. The performance of the UNet network is also relatively good with an ACC value of 0.99. The MSRN network has the least params among all methods with only 12.42 M, and it also has a recall of 0.945. Comparatively, although the MSRN network also requires only 24.53 M parameters, it has significantly lower DSC values. For the PSPNet algorithm, it has the lowest FLOPS value but it has the worst IOU and DSC values. It indicates that the performance of this model is poor. Although our method has more parameters than UNet, MSFCN, and MSRN models, it has fewer parameters than some other convolution-based models. It has higher segmentation accuracy and faster computation speed with only a small number of additional parameters.

**Figure 11:**
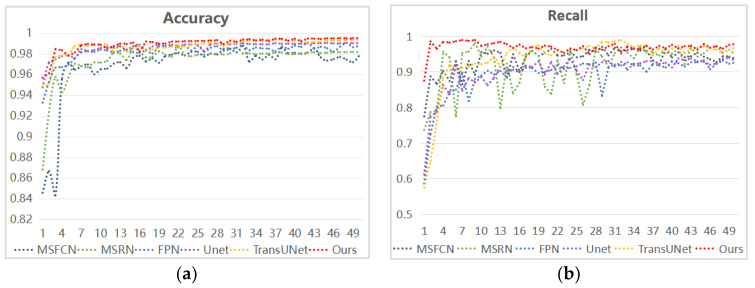

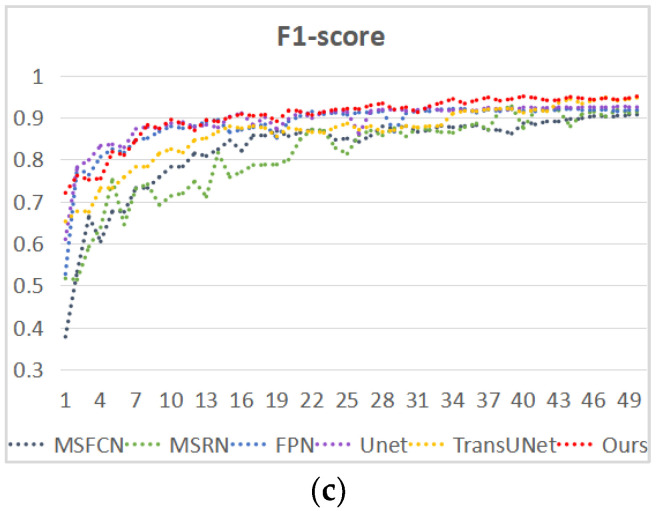
The trend of different metrics of each network during the training process. (**a**) is the trend of model accuracy, (**b**) is the trend of model recall, and (**c**) is the trend of model F1-score values.

Each model was trained for a total of 200 rounds, and 1 round was selected every 4 rounds for visualization. As can be seen from Figure 11, our method is the fastest to reach the best stability in terms of accuracy, recall, and F1 value. In Figure 11a, the accuracy ranking among the models is ours > TransUNet > Unet > FPN > MSRN > MSFCN. In Figure 11b, overall, the recall rate of our suggested method has been kept as high as possible, ensuring that missed diagnoses are avoided to the greatest extent possible. Finally, the F1-score of each model in the training process is compared with our method. From Figure 11c, we can see that our F1 value fluctuates to some extent, but its average value remains the highest. It can be seen that the model is more robust and accurate in processing MRI images of osteosarcoma with different morphological characteristics.

### 4.6. Discussion

It is shown that the pre-screened TSF shows a robust and accurate classification capability, effectively filters redundant data, and improves segmentation performance while saving the running time of subsequent processing operations. The fast FFT-based NLM algorithm effectively improves image segmentation and largely reduces the impact of noise in osteosarcoma diagnosis. 

We designed TBNet, a U-Net network with a fusion transformer and edge enhancement, for the osteosarcoma MRI Image segmentation problem. Several comparison studies reveal that our model outperforms others in terms of assessment indices such as DSC and IOU. It offers significant benefits over other classical convolutional models for the proper processing of contour features in the complicated tumor border problem. In addition, comparative tests in terms of accuracy, recall, and F1 by systematic sampling of random samples show that our model has greater stability. This ensures the stability and accuracy of segmentation results for practical diagnosis. This is mainly due to the introduction of a cross-fusion channel transformer with edge-enhancement features to replace the original U-Net jump connection in this paper. This approach takes advantage of both a transformer and Unet to perform the cross-fusion of multi-scale channel information, bridge the semantic gap, and solve the problem of semantic hierarchy inconsistency. In addition, the algorithm improves the problem of fuzzy edge segmentation by enhancing the edge information. Therefore, compared with the classical UNet model, the recognition accuracy of our method is higher. To apply to developing countries, our model reduces the computational cost as much as possible while ensuring accuracy and stability.

## 5. Conclusions

In this study, an artificial intelligence-aided diagnosis system for osteosarcoma MRI pictures with edge-enhancement characteristics is suggested. The method employs strategies such as image pre-screening, noise-reduction processing, and a segmentation model using edge-enhancement characteristics. It is used mainly to improve the recognition accuracy of osteosarcoma and assist doctors to detect the lesion location of patients more quickly and accurately. Our model is more robust in processing the MRI images of osteosarcoma with different shapes. 

However, the recognition accuracy of the TBNet model is relatively low for images without noise reduction and pre-screening. With the development of image processing technology, we will strive to improve the recognition accuracy of tumors in original images. In addition, improving the function of the auxiliary scheme in combination with the actual clinical performance is also the focus of research.

## Figures and Tables

**Table 1:** Main mathematical notations and their annotations.

Notation	Meaning
${\mathbb{Z}}^{2}$	The osteosarcoma MRI image domain
$B_{a}$	$A\;\mathrm{block}\;\mathrm{of}\;\mathrm{image}\;\mathrm{windows}\;\mathrm{of}\;\mathrm{size}\;n_{1}\times n_{1}$
${\bar{B}}_{a}$	$\mathrm{The}\;\mathrm{result}\;\mathrm{of}\;B_{a}\;$initialized noise reduction
$\lambda_{T}$	The hyperparameter of the TSF
p	The Euclidean distance
$\lambda_{FFT2}$	The fixed threshold parameter
X	MR noise data
S	Original MR images and Phases
k(a)	Noise component intensity of the i-th pixel
$I_{0}$	The modified Bessel function
$\eta_{1},\eta_{2}$	Real and virtual channel impact
k(a)	The activation function of TSF
$\omega (N_{i},N_{j}$)	The function of weighted similarity
$F_{2D},F_{2D}^{-1}$	The DCT transform operator (FFT) and its inverse
Z(i)	The normalization constant
$\phi \left(\cdot \right)$	The boundary level set
$C_{S}$	Search window side half-length
$M_{i},{\hat{M}}_{i}$	The similarity matrix and Mask edge diagram
$\psi \left(\cdot \right)$	The instance normalization
$\delta \left(\cdot \right)$	The ReLU operator
$f_{i-1}$	Supplementary layer feature map for layer *i* − 1
$g\left(\cdot \right)$	The probabilistic output of the network

**Table 2:** Analytical constant images of TSF algorithm screening results.

	True	Lesion Image	Normal Image
Predicted			
Lesion image		49	4
Normal image		3	44

**Table 3:** Comparison of TBNet under different conditions.

Model	ACC	IOU	DSC	Pre	Re	F1
TBNet	0.991	0.904	0.931	0.927	0.974	0.949
TBNet+Denoise	0.993	0.915	0.943	0.932	0.968	0.951
TBNet+Denoise+Pre-Screening	0.997	0.915	0.949	0.941	0.969	0.954

**Table 4:** Performance comparison of different models on the osteosarcoma task.

Model	ACC	IOU	DSC	Pre	Re	F1	FLOPS	Params
FCN-16s	0.989	0.824	0.859	0.922	0.882	0.900	187.35 G	122.4 M
FCN-8s	0.993	0.830	0.876	0.941	0.873	0.901	187.18 G	122.4 M
PSPNet	0.975	0.772	0.870	0.856	0.888	0.872	103.55 G	47.70 M
MSFCN	0.991	0.841	0.874	0.881	0.936	0.906	1642.43 G	24.53 M
MSRN	0.988	0.853	0.887	0.893	0.945	0.918	1346.12 G	12.42 M
FPN	0.989	0.852	0.888	0.914	0.924	0.919	134.14 G	47.82 M
UNet	0.990	0.867	0.892	0.922	0.924	0.923	160.16 G	17.26 M
UTNet	0.990	0.879	0.919	0.924	0.934	0.936	264.27 G	52.53 M
TransUNet	0.993	0.898	0.923	0.919	0.959	0.948	240.26 G	40.38 M
Our(TBNet)+Denoise+Pre-Screening	0.997	0.915	0.949	0.941	0.969	0.954	235.26 G	36.51 M

## Data Availability

All data analyzed during the current study are included in the submission. Data used to support the findings of this study are currently under embargo while the research findings are commercialized. Requests for data, 12 months after the publication of this article, will be considered by the corresponding author.
